# Factors Affecting Compliance with Clinical Practice Guidelines for Pap Smear Screening among Healthcare Providers in Africa: Systematic Review and Meta-Summary of 2045 Individuals

**DOI:** 10.1371/journal.pone.0072712

**Published:** 2013-09-12

**Authors:** Etienne Asonganyi, Meenakshi Vaghasia, Clarissa Rodrigues, Amruta Phadtare, Anne Ford, Ricardo Pietrobon, Julius Atashili, Catherine Lynch

**Affiliations:** 1 Maternity Unit, Kumba District Hospital, Kumba, Southwest Province, Cameroon; 2 Department of Surgery, Duke University, Durham, North Carolina, United States of America; 3 Instituto de Cardiologia do RS - Fundação Universitária de Cardiologia, Porto Alegre, Rio Grande do Sul, Brazil; 4 Duke Women’s Health Associates, Duke University, Durham, North Carolina, United States of America; 5 Department of Epidemiology, University of Buea, Buea, Southwest Region, Cameroon; 6 Division of Emergency Medicine, Duke School of Medicine and Duke Global Health Institute, Duke University, Durham, North Carolina, United States of America; The University of Hong Kong, Queen Mary Hospital, Hong Kong

## Abstract

**Background:**

Although the importance of the Pap smear in reducing cancer incidence and mortality is known, many countries in Africa have not initiated yet widespread national cervical cancer screening programs. The World Health Organization (WHO) has published Clinical Practice Guidelines (CPGs) on cervical cancer screening in developing countries; however, there is a gap between expectations and clinical performance. Thus, the aim of this study was to conduct a systematic review and meta-summary to identify factors affecting compliance with CPGs for Pap screening among healthcare providers in Africa.

**Methods:**

**And Findings**: MEDLINE, Scirus, Opengate and EMBASE databases were searched in January 2012. Studies involving medical personnel practicing in Africa, whose outcome measured any factors that affect medical personnel from using a Pap smear to screen for cervical cancer, were included. Two reviewers independently evaluated titles and abstracts, then full-texts, extracted data and assessed quality of the included studies. A descriptive analysis of the included studies was conducted. We calculated Frequency effect sizes (FES) for each finding and Intensity effect sizes (IES) for each article to represent their magnitudes in the analyses. Of 1011 studies retrieved, 11 studies were included (2045 individuals). Six different themes related to the factors affecting compliance with CPGs were identified: Insufficient Knowledge/Lack of awareness (FES = 82%), Negligence/Misbeliefs (FES = 82%), Psychological Reasons (FES = 73%), Time/Cost Constraint (FES = 36%), Insufficient infrastructure/training (FES = 45%) and also no reason given (FES = 36%). IES for articles ranged between 33 and 83%.

**Conclusions:**

These results suggest that prevention initiatives should be comprehensive to include education and resources needs assessments and improvement, Pap smear test training, strategies on costing, and practitioner time studies.

## Introduction

Cervical cancer is the second most common cancer among women worldwide, and the leading cause of cancer-related deaths among women in developing countries. In 2008, 453,000 new cases and 242,000 deaths occurred from cervical cancer, with 83% of the cases of cervical cancer occurring in developing countries [1,2]. Although the effectiveness of the Pap smear in reducing cervical cancer incidence and mortality has already been demonstrated in many developed countries [3,4], there is a wide disparity in rates of screening for cervical cancer in developing countries with the average screening coverage rate in developed countries at 63% compared to 19% in developing countries [5]. In the developing world, women at highest risk for developing cervical cancer are among the least likely to be screened [5]. In spite of the World Health Organization (WHO) published clinical practice guidelines (CPGs) on cervical cancer screening in developing countries, most developing African countries have not initiated widespread national cervical cancer screening programs. A WHO report on cervical cancer screening in Sub-Saharan Africa noted that while this region was the most affected by cervical cancer, it has access to less than 5% of the global resources for cervical cancer prevention [6]. Additionally, health system strengthening, in order to promote, restore, or maintain health, requires the translation of WHO guidelines into national guidelines and the development of implementation strategies [7].

In African countries, not only is the incidence of cervical cancer high but a large proportion of patients present with advanced disease (> stage IIb) [8,9]. These data suggest that in African countries there is a lack of a successful large scale screening programs. Despite the significant lack of screening on the health of women in Africa, there have been no previous systematic reviews to identify the factors leading to compliance or non-compliance with CPGs on Pap smear testing for cervical cancer screening in Africa.

Physician behavior, specialty and gender have all shown to play an important role in patient compliance [10,11]. In a systematic review of physician non-adherence to general clinical practice guidelines, the authors noted many factors could limit adherence like lack of awareness, familiarity, agreement, self-efficacy, and outcome expectancy as well as inertia of previous practice. However, the authors emphasize that, since barriers may differ from setting to setting, these results may not be generalizable [12].

In Africa, factors that have been shown to affect physicians’ compliance include but are not limited to: busy clinics and lack of manpower, lack of access to care, lack of transport to care and opposition to care by men [13]. Another review investigating guideline compliance concluded that there is a considerable gap between expectations and clinical performance but did not investigate the reasons for this disparity [14]. These studies did not address the compliance with clinical guidelines on Pap smear testing. The objective of this systematic review is to identify factors affecting compliance with Clinical Practice Guidelines for Pap screening among healthcare providers in Africa*.*

**Table 1:** Characteristics of the included studies.

Reference	Country	Study Design	No of Individuals	Occupation	Gender	Age
Mutyaba T. , et al (2006) [20]	Uganda	Descriptive Cross-Sectional Survey	288	Medical Officers = 39 (13.5%), Nurses = 167 (58.0%), Specialists = 19 (6.6%), Students = 63 (21.9%)	198 Females (69%)	*
Dim CC. , et al (2009) [21]	Nigeria	Survey Questionnaires	79	Fellows = 17 (22%), Senior Residents = 16 (20%), Junior Residents = 17 (22%), Medical Officers = 14 (18%), Interns = 15 (19%)	Females	24-59 years, mean of 35.9±8 years
Udigwe GO. , et al (2006) [22]	Nigeria	Self-administrated Questionnaire Survey	140	Nurses	Females	20-29 years = l 6 (11.4%), 30-39 years = 80 (57.2%), 40-49 years = 34 (24.3%), 50-59 years = 9 (6.4%), ≳60 years = 1 (0.7%)
Gharoro EP. , et al (2006) [23]	Nigeria	Survey	184	Doctors = 16 (8.7%), Nurses = 109 (59.2%), Pharmacists = 4 (2.2%), Lab. Technicians = 4 (2.2%), Hospital Maids = 41 (22.3%), Radiographers = 3 (1.6%), Others = 7 (3.8%)	Females	24-60 years, mean 39.6±7.3 years
Nwobodo EI. , et al (2005) [24]	Nigeria	Cross-Sectional Survey	159	Doctors = 18 (11.3%), Nurses = 127 (79.9%), Pharmacists = 4 (2.5%), Lab. Scientists = 7 (4.4%), Social Workers = 3 (1.9%)	Females	24-53 years, mean of 34.2±6.8 years
Anya SE. , et al (2005) [25]	Nigeria	Questionnaire Survey	144	Doctors = 21 (14.6%), Nurses = 76 (52.8%), Pharmacists = 20 (13.9%), Lab. Scientists = 27 (18.8%)	Females	20-29 years = 41 (28.5%), 30-39 years = 50 (34.7%), 40-49 years = 46 (31.9%), σ50 years = 7 (4.9%)
Tarwireyi F. , et al (2003) [26]	Zimbabwe	Cross-Sectional Survey	60	Doctors = 1 (1.7%), Nurses = 34 (56.7%), Nurses Aide = 22 (36.6%), Allied = 3 (5.0%)	41 Females (68.3%)	20-29 years = 9 (15.0%), 30-39 years = 37 (61.7%), σ 40 years = 14 (23.3%), Mean of 33±7 years
Aboyeji PA. , et al (2004) [27]	Nigeria	Cross-Sectional Survey	483	Nurses = 405 (83.9%), Doctors = 31 (6.4%), Pharmacists = 12 (2.5%), Lab. Scientists = 23 (4.8%)	Females	20-24 years = 7, 25-29 years = 56, 30-34 years = 104, 35-39 years = 98, σ 40 years = 218
Olaniyan OB. , et al (2000) [28]	Nigeria	Cross-Sectional Survey	166	Doctors = 15 (9.0%), Nurses = 118 (71.1%), Pharmacists = 10 (6.0%), Lab. Scientists = 17 (10.2%), Social Workers = 6 (3.6%)	Females	Mean of 33.9±6.1 years
Urasa M. , et al (2011) [29]	Tanzania	Descriptive Cross-Sectional Study	137	Enrolled Nurses = 70 (51.0%), Registered Nurses = 67 (49.0%)	Females	< 30 years = 8 (5.8%), 30-40 years = 44 (32.1%), > 40 years = 85 (62%), Mean of 44.2±9.3 years
Ayinde OA. , et al (2003) [30]	Nigeria	Survey	205	Doctors = 45 (22.0%), Nurses = 90 (43.9%), Hospital Maids = 70 (34.1%)	Females	< 20 years = 2 (1%), 20-40 years = 143 (69.8%), > 40 years = 60 (29.2%)

* Study did not provide the data.

## Methods

### Protocol and Registration

This systematic review is reported in accordance with the Preferred Reporting Items for Systematic Review and Meta-Analyses (PRISMA) statement [15]. The protocol of the study can be found in File S1, and the PRISMA check list in Table S1.

### Eligibility Criteria

The following inclusion criteria were considered: 1) Studies involving medical personnel; 2) Studies whose outcome measures included any factors that affect medical personnel from using a Pap smear to screen for cervical cancer, 3) Studies conducted in Africa, and 4) Observational design studies. We excluded studies that retrospectively analyzed clinical trial data, unpublished articles, dissertations, and abstracts without full text. In addition, manuscripts in languages other than English, French or Portuguese were not included in the review.

### Information Sources

We searched the following electronic databases for published literature up until January 2012: PubMed, Scirus, Opengate, Directory of Open Access Journals (DOAJ), and EMBASE. We did not use limits for language when searching the databases. The references of the included articles were reviewed, as well as we performed citation analysis of the included studies using Google Scholar, and also sought experts’ suggestions through email communications.

**Table 2:** Risk of bias assessment.

Articles and Assessment Criteria	Mutyaba T. , et al (2006) [20]	Dim CC. , et al (2009) [21]	Udigwe GO. , et al (2006) [22]	Gharoro EP. , et al (2006) [23]	Nwobodo EI. , et al (2005) [24]	Anya SE. , et al (2005) [25]	Tarwireyi F. , et al (2003) [26]	Aboyeji PA. , et al (2004) [27]	Olaniyan OB. , et al (2000) [28]	Urasa M. , et al (2011) [29]	Ayinde OA. , et al (2003) [30]
Are the criteria for inclusion of subjects described?	Yes	Yes	Yes	Yes	Yes	Yes	Unclear	Yes	Yes	Yes	Yes
Has the study sample been clearly described in terms of sample size and demographic characteristics such as age, race, gender, location, socioeconomic status, etc?	No	Yes	Yes	Yes	No	Yes	Yes	Yes	Yes	Yes	Yes
Is the study sample appropriate to the problem being studies or the hypotheses being tested?	Yes	Yes	Yes	Yes	Yes	Yes	Yes	Yes	Yes	Yes	Yes
Is the study sample large enough to test the hypotheses?	Unclear	Unclear	Unclear	Yes	Yes	Unclear	Unclear	Yes	Unclear	Yes	Unclear
How was the study sample selected (random, haphazard, consecutive patients presenting with a particular disease, all subjects in a particular group, etc)	Yes	Yes	Yes	Yes	Yes	Yes	Unclear	Yes	Unclear	Yes	Unclear
Is the design of the study clearly described?	Yes	Yes	Yes	Yes	Yes	Yes	Yes	Yes	Yes	Yes	Yes
Does the design of the study adequately test the hypotheses?	Yes	Yes	Yes	Yes	Yes	Yes	Yes	Yes	Yes	Yes	Yes
How was random selection of subjects achieved? Was any other method besides the use of random numbers table used?	No	Yes	No	No	No	No	No	Unclear	No	Yes	Unclear
Have the measurement of the outcome, independent, and control variables been clearly described?	Yes	Yes	No	Yes	No	Yes	No	Unclear	Unclear	Yes	Yes
Are the variables measured with appropriate and accurate methods? Do the operational definitions match the theoretical variables?	Yes	Unclear	Unclear	Yes	Unclear	Yes	Unclear	Unclear	Unclear	Yes	Yes
Have the laboratory tests, instruments and/or questionnaires used to measure the variables undergone validity and reliability testing?	No	No	No	No	No	No	No	Unclear	Unclear	Unclear	No
Have the procedures or methods undergone standardization for a particular population that is being studied?	No	No	No	No	No	No	No	Unclear	Unclear	Unclear	No
Were the outcomes variables measured using appropriate blinded methods?	No	No	No	No	No	No	No	No	No	No	Yes
Have the number of non-respondents, refusals, and subjects lost the follow-up been kept reasonably small (less than 10%)	Yes	Yes	No	Yes	Unclear	Yes	Yes	Yes	Unclear	Yes	Unclear
Was there strict adherence to the protocol?	Yes	Yes	Yes	Yes	Unclear	Yes	Yes	Yes	Unclear	Yes	Unclear

Yes – Low risk of bias

Unclear – Moderate risk of bias

No – High risk of bias.

### Search

The initial search comprised the following Mesh terms "*Vaginal Smears*", *and* "*Africa*" and the related entry terms. The complete search strategy used for the PubMed database is shown in Table S2.

### Study Selection

Titles and abstracts of the retrieved articles were independently evaluated by two reviewers (E.A. and M.V.). Abstracts that did not provide enough information regarding the eligibility criteria were kept for full-text evaluation. Reviewers independently evaluated full-text articles and determined study eligibility. Disagreements were solved by consensus and if disagreement persisted, we sought a third reviewer’s opinion (A.J.).

### Quality of Studies

Several tools have been proposed for evaluation of methodological quality of observational epidemiological studies [16]. In this study, quality assessment was based on a checklist specific for evaluating cross-sectional studies [17], which is based on 15 items. We chose this checklist based on a systematic review previously published [18], which recommends to use checklists rather than scales, as well as to use a tool as specific as possible, considering study design and area.

### Data Extraction

Two reviewers (E.A. and M.V.) independently conducted data extraction and disagreements were also solved by the third reviewer (A.J.). The data extraction spreadsheet was pre-tested by the two reviewers and alterations were made as necessary. General characteristics of the studies were collected, such as: year of publication, location and setting where the study took place, number of health care providers, health providers characteristics. In addition, we collected from each study the factors cited as affecting compliance with CPGs for Pap smear screening among healthcare providers in Africa.

### Data Analysis

We performed a descriptive analysis of factors affecting healthcare provider compliance with CPGs for Pap smear in Africa, described in the included studies. Factors affecting compliance were grouped by similarity, and within each group, brief descriptions of the findings were generated. To represent the magnitude of each finding, we adapted the methodology proposed by Sandelowski M, et al (2007) [19]. Frequency effect sizes (FES) were generated by dividing the number of studies citing a particular theme by the total number of studies in our final list and multiplied by 100. The themes with higher FES are the ones more prevalent across the included papers. To represent the magnitude of each report, intensity effect sizes (IES) were calculated. Specifically, for each study, the number of findings with a FES >25% was divided by the total number of findings with frequency effect size >25%. Additionally, for each study, the number of themes it cited was divided by the total number of themes overall [19]. The studies with higher IES are the ones presenting more themes. As the lowest FES identified was 36%, and both IES were the same, we report only one value for IES for each study.

**Figure 1:**
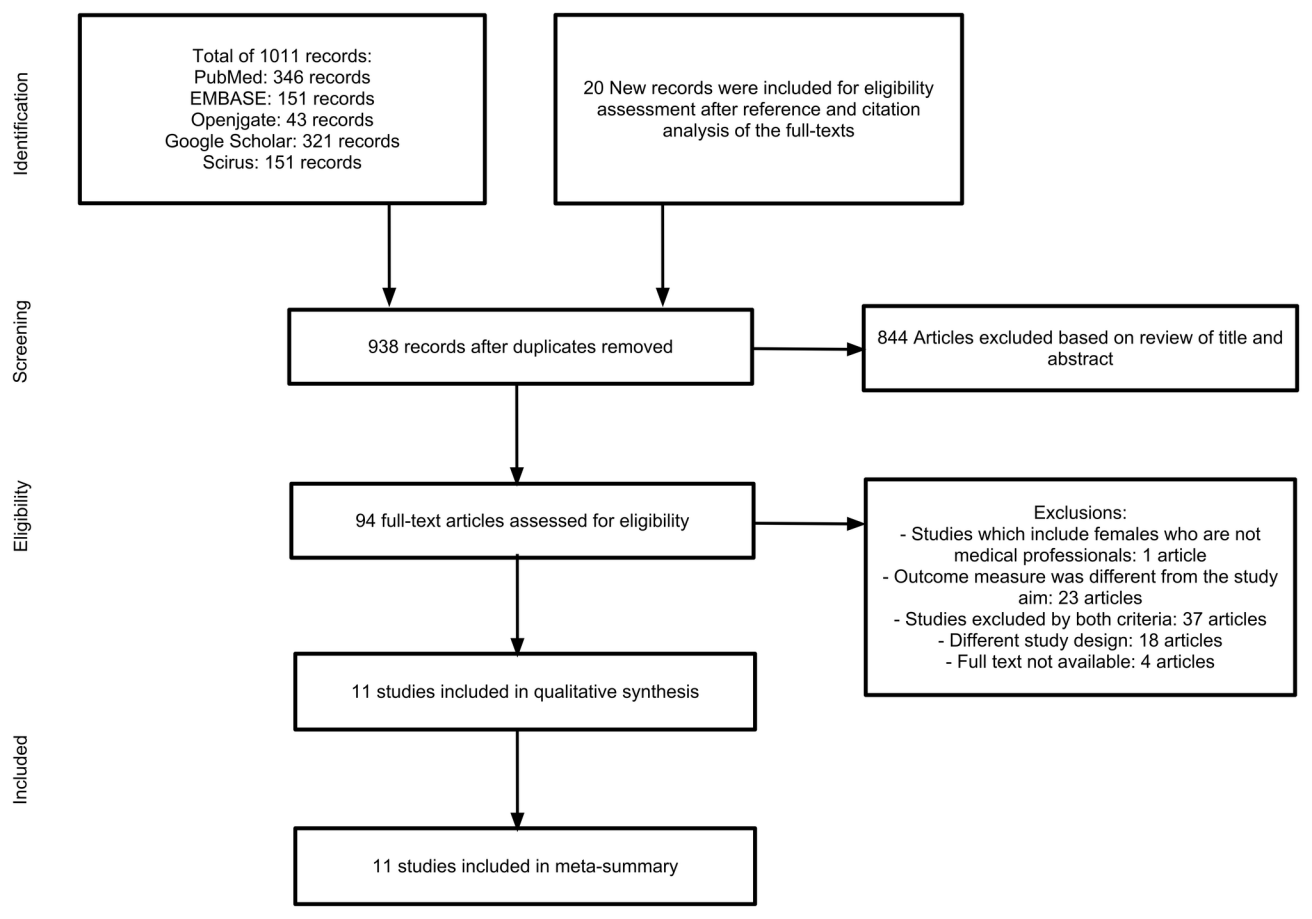
PRISMA flowchart.

## Results

### Characteristics of included studies

A total of 1011 records were identified and screened with 94 full-text articles assessed for eligibility based on the inclusion and exclusion criteria. Ultimately, 11 surveys were included in the qualitative synthesis and meta-summary. Figure 1 illustrates the search and article selection process as well as the numbers of articles retrieved and included during the article selection phases of this project.

**Table 3:** Factors affecting compliance with CPGs for Pap smear screening identified in each study.

Reference	Studies Findings	Themes Identified
Mutyaba T. , et al (2006) [20]	Knowledge: 81% had knowledge about whether cervical cancer is curable, 29% had knowledge about risk factors for cervical cancer, 81% had knowledge about Pap smear screening, 26% had knowledge about eligibility for screening, and 39% had knowledge about cancer screening interval. Attitudes: 93% thought cancer of the cervix was a public health concern, 68% thought that it was easy to diagnose, 65% of the participant females did not think they were susceptible to cervical cancer themselves, 60% of males thought that their partners were susceptible. Most nurses and midwives thought that speculum examination and Pap smear were doctors’ procedures, 22% of the medical students thought they were for senior doctors only, doctors in disciplines other than gynecology thought that speculum examination was an activity for gynecologists only, lack of vaginal specula and absence of indication for speculum examination were common reasons for not screening patients, among the females respondents, reasons for not having been screened included: not feeling at risk, lack of symptoms, carelessness, fear of vaginal examination, lack of interest, test being unpleasant and not yet being of risky age. Moreover, 25% of the female respondents said that they would only accept a vaginal examination by a female health worker. Medical students were asked for strongest reason for not performing Pap smears. Responses were: 35% thought they were not allowed, 15% never thought about it, 22% thought it was for senior doctors and 26% did not know how to do one. Practices: routine management of female patients -86%, frequently performing vaginal examinations -62%, speculum use during vaginal examinations -12%, females respondents who have ever been screened themselves -19%, male respondents whose partners have ever been screened -26%, don’t ask patients whether screened -78%, and don’t refer patients for screening -78%.	Insufficient Knowledge/Lack of Awareness, Negligence/Misbeliefs Psychological Reasons, Insufficient infrastructure/training
Dim CC. , et al (2009) [21]	Reasons for non-use of Pap smear by female medical practitioners: poor health consciousness -2 (3%), do not feel susceptible to cervical cancer -6 (9%), scared of the outcome -4 (6%), too busy to screen -15 (23%), just lazy about screening -15 (23%), preservation of virginity -1 (2%), awaiting menopause -1 (2%), no reason -21 (23%), and non-accessibility of Pap smear – zero.	Insufficient Knowledge/Lack of Awareness, Negligence/Misbeliefs Psychological Reasons, Time/Cost constraint, No reason given
Udigwe GO. , et al (2006) [22]	Reasons for not undergoing Pap smear: ignorance of availability -26 (18.6%), fear of outcome -21 (15.0%), not a likely candidate -35 (25.0%), financial implication -1 (0.7%), no reason -52 (37.1%), and not applicable -8 (5.7%).	Insufficient Knowledge/Lack of Awareness, Negligence/Misbeliefs Psychological Reasons, Time/Cost constraint, No reason given
Gharoro EP. , et al (2006) [23]	More than 65% of the respondents were aware of the disease, cervical cancer, and approximately 64% were aware of the Pap smear test. Pap smear awareness level signiﬁcantly varied among the categories of the female health workers. A minority of 14.1% has had a Pap smear test. There was a signiﬁcant variation in utilization of Pap smear test across the various categories of the health workers and a signiﬁcant correlation between Pap smear awareness and utilization. The majority, 89%, believed that they were not at risk of developing cervical cancer. The self-reported utilization of Pap smear test among health workers was low. While there was a positive correlation between Pap smear test awareness and utilization, screening uptake was very poor due to a combination of inappropriate beliefs, misapprehension, and deﬁcient knowledge.	Insufficient Knowledge/Lack of Awareness, Negligence/Misbeliefs Psychological Reasons, Insufficient infrastructure/training
Nwobodo EI. , et al (2005) [24]	Reasons for not having Pap smear: no physician referral -98 (64.5%), did not feel susceptible to cancer of the cervix -25 (16.4%), did not believe in the test -7 (4.6%), have no knowledge of Pap smear -5 (3.3%), did not know where to have the test -5 (3.3%), fear of the result -3 (2.0%), and no reason given -9 (5.9%).	Insufficient Knowledge/Lack of Awareness, Negligence/Misbeliefs, Psychological Reasons
Anya SE. , et al (2005) [25]	Knowledge of cervical cancer and Pap smear: 91.7% had heard of cervical cancer while 80.6% knew it was associated with abnormal vaginal bleeding, 22.2% could not list any risk factor for cervical cancer, 32.6% believed it was potentially curable and 70.8% that it could be prevented, and 77.8% reported they had heard of Pap smear.	Negligence/Misbeliefs, Psychological Reasons, Time/Cost constraint, Insufficient infrastructure/training
	Attitudes: 88.6% who had heard of cervical cancer considered it a serious problem, 89 respondents who knew the purpose of a Pap smear, 92.1% would recommend regular Pap smear if these were affordable, only 9% had ever had a Pap smear, profession and marital status were the two determinants of likelihood to have had a Pap smear. Doctors and divorced/separated women were more likely to take up Pap smears. Reasons for non-uptake of Pap smears among those knew its purpose: not available -39 (51.3%), have not thought of it -17 (22.4%), cannot afford it -14 (18.4%), and no personal risk of cervical cancer -6 (7.0%).	
Tarwireyi F. , et al (2003) [26]	Knowledge of risk factors: early sexual intercourse -21 (35%), using vaginal herbs and chemicals -51 (85%), infection by the human papilloma virus -17 (28.3%), HIV infection -6 (10%), multiple sexual partners -26 (43.3%), and multiple pregnancies -8 (13.3%). Knowledge of pre-cervical cancer treatments options: cryotherapy -17 (28.3%), knife cane biopsy -9 (15.0%), electro-diathermy -11 (18.3%), leep – zero, and laser – zero.	Insufficient Knowledge/Lack of Awareness, Insufficient infrastructure/training
Aboyeji PA. , et al (2004) [27]	Reasons for not wanting to be screened: cannot have cervical cancer -137 (52.5%), fear of detecting of cervical cancer -50 (19.2%), screening against religious belief -38 (14.6%), screening expense -35 (13.4%), my husband is against it -27 (10.3%), and no particular reason -34 (13.0%).	Negligence/Misbeliefs Psychological Reasons, Time/Cost constraint, No reason given
Olaniyan OB. , et al (2000) [28]	Reasons for not having Pap Smear: no physician referral -57 (54.3%), did not feel susceptible to cancer -27 (25.7%), did not know where to have the test -6 (5.7%), would require husband’s permission -2 (1.9%), did not believe in test -2 (1.9%), and no reason given -6 (5.7%).	Insufficient Knowledge/Lack of Awareness, Negligence/Misbeliefs Psychological Reasons, No reason given
Urasa M. , et al (2011) [29]	Knowledge of causes of HPV: HPV infection -53 (38.7%), genetic predisposition -32 (23.4%), certain foods -131 (95.6%), and bacterial infectious -103 (75.2%). Knowledge of Transmission of HPV: sexual intercourse -83 (60.6%), direct genital contact -38 (27.7%), kissing -137 (100%), body fluids -112 (81.8%), drinking unsafe water -135 (98.5%), mother to child transmission -130 (94.9%), and air droplets -136 (99.3%). Knowledge of risks of cervical cancer: smoking -28 (20.4%), alcohol -123 (89.8%), multiple sexual partners -65 (47.4%), history of HPV infection -60 (43.8%), early sexual debut -51 (37.2%), impaired immunity -11 (8.0%), use of Intrauterine device -110 (86.9%), and poor hygiene -136 (99.3%). Knowledge of symptoms of cervical cancer: post-coital bleeding -63 (46%), inter-menstrual bleeding -13 (9.5%), blood stained vaginal discharge -73 (53.3%), fever -134 (97.8%), headache -136 (99.3%), pelvic pain -26 (38%), post-menopausal bleeding -52 (38%), and painful coitus -59 (43.1%).	Insufficient Knowledge/Lack of Awareness, Insufficient infrastructure/training
Ayinde OA. , et al (2003) [30]	Knowledge about cancer of the cervix was highest among doctors, followed by nurses and hospital maids, consecutively. Only 6.8% had a previous Pap smear. Reasons for not having Pap smear in those who have never had it: cost consideration -13 (6.8%), lack of awareness about test -83 (43.5%), lack of awareness about locations where the test is performed -17 (8.9%), reluctance -67 (35.1%), not yet sexually exposed -6 (3.1%), belief in not being prone to cervical cancer -5 (2.6%).	Insufficient Knowledge/Lack of Awareness, Negligence/Misbeliefs Psychological Reasons

**Table 4:** Themes identified in the included studies and the respective Frequency effect sizes (FES).

Themes	No of Studies - FÈS (%)*	Reference
Insufficient Knowledge/ Lack of awareness	9 (82)	Mutyaba, et al. (2006) [20], Dim CC, et al (2009) [21], Udigwe GO, et al (2006) [22], Gharoro, et al. (2006) [23], Nwobodo, et al. (2005) [24], Tarwireyi F, et al (2003) [26], Olaniyan OB, et al. (2000) [28], Urasa M, et al. (2011) [29], Ayinde, et al. (2003) [30]
Negligence/ Misbeliefs	9 (82)	Mutyaba, et al. (2006) [20], Dim CC, et al (2009) [21], Udigwe GO, et al (2006) [22], Gharoro, et al. (2006) [23], Nwobodo, et al. (2005) [24], Anya SE. , et al (2005) [25], Aboyeji PA. , et al (2004) [27], Olaniyan, et al. (2000) [28], Ayinde, et al. (2003) [30]
Psychosocial Reasons	8 (73)	Mutyaba, et al. (2006) [20], Dim CC, et al (2009) [21], Udigwe GO, et al (2006) [22], Gharoro, et al. (2006) [23], Nwobodo, et al. (2005) [24], Aboyeji PA. , et al (2004) [27], Olaniyan, et al. (2000) [28], Ayinde, et al. (2003) [30]
Time/Cost Constraint	4 (36)	Dim, et al. (2009) [21], Udigwe, et al. (2006) [22], Anya, et al. (2005) [25], Aboyeji, et al. (2005) [27]
Insufficient infrastructure/ training	5 (45)	Mutyaba T. , et al (2006) [20], Gharoro, et al. (2006) [23], Anya, et al. (2005) [25], Tarwiyeri, et al. (2003) [26], Urasa M, et al. (2011) [29]
No reason given	4 (36)	Dim, et al. (2009) [21], Udigwe, et al. (2006) [22], Aboyeji PA. , et al (2004) [27], Olaniyan, et al. (2000) [28]

* FES were computed by taking the number of reports containing a finding (minus any reports derived from a common parent study and representing a duplication of the same finding) and dividing this number by the total number of reports (minus any reports derived from a common parent study and representing a duplication of the same finding).

### Studies Characteristics

Included studies had a total of 2045 individuals. Sample sizes varied from 60 to 483 with a mean of 185.9 of which two studies included both females and male respondents. The characteristics for the studies included in this analysis are shown in Table 1.

### Quality of Studies

Table 2 demonstrates the risk of bias assessment of the included studies. There was prevalence of “Yes“, which means low risk of bias (93/165), however many studies were ‘Unclear’ in their presentation of some items, representing moderate risk of bias (33/165). Also, some studies did not present come items at all (“No“), being classified as high risk of bias (39/165).

**Table 5:** Intensity effect sizes (IES) for each report.

Reference	IES*
Mutyaba T. , et al (2006) [20]	67
Dim CC. , et al (2009) [21]	83
Udigwe GO. , et al (2006) [22]	83
Gharoro EP. , et al (2006) [23]	67
Nwobodo EI. , et al (2005) [24]	50
Anya SE. , et al (2005) [25]	67
Tarwireyi F. , et al (2003) [26]	33
Aboyeji PA. , et al (2004) [27]	67
Olaniyan OB. , et al (2000) [28]	67
Urasa M. , et al (2011) [29]	33
Ayinde OA. , et al (2003) [30]	50

* IES was derived by dividing the number of findings contained in that report by the total number of findings across all reports.

### Synthesis of Results

Factors affecting compliance with CPGs for Pap smear screening identified in the articles are presented in Table 3. The systematic review uncovered multiple main themes for factors affecting non-compliance with CPGs for Pap screen including: insufficient knowledge/awareness, negligence/misbeliefs, psychosocial reasons, time/cost constraints and insufficient infrastructure.

The results of the meta-summary demonstrated all findings reported a FES > 25, ranging range from 82% to a low of 36%. Effect sizes greater than 25% are considered more relevant. Non-compliance factors had the highest FES, meaning the most common findings amongst studies were Insufficient Knowledge/Lack of Awareness and Negligence/Misbeliefs. Each of these themes and their corresponding FES are displayed in Table 4.

The IES for each of the included articles is displayed in Table 5. The results demonstrate IES ranging from 50 to 83%. The study of Olaniyan OB, et al (2002) contains the largest number of findings, in contrast to the study of Tarwireyi F, et al (2003), which had the lowest number of findings.

## Discussion

To our knowledge, this is the first systematic review describing the factors that affect compliance with CPGs for Pap smear screening among healthcare providers in Africa. Our review identified 11 studies that cited factors for noncompliance with Pap smear protocols including Insufficient Knowledge/Lack of Awareness, Negligence/Misbeliefs, Psychological Reasons, Time/Cost Constraints and Insufficient Infrastructure/Training.

The factors found in our study to be most cited and therefore that had the highest FES were Insufficient Knowledge/Lack of Awareness and Negligence/Misbelief for not complying with Pap smear guidelines. Surprisingly, even among professionals with knowledge on Pap smear and cervical cancer, most of them have never had a Pap smear. This result is in agreement with another study that found that improved awareness of Pap smear may not affect its use in Nigeria, in which all respondents were aware of the Pap smear but only 18% had used it before [31]. In South Africa, a national study evaluating 20,603 women in public health service around the country found that 80% had never had a Pap smear [32]. In this South African context, barriers to effective screening programs are further complicated by the legacy of racial and geographic inequity in education and literacy levels, health infrastructure and access to health services, thus representing a considerable challenge [33].

Previous studies have evaluated cervical cancer and Pap smear awareness in different populations [34-42]. A recent study conducted in Nigeria aimed to determine the level of awareness of cervical cancer and Pap smear test, and factors associated with the utilization of Pap test among female civil servants in Jos. In this study, 51% of participants had cervical cancer awareness while 39% were aware of Pap smear testing. In addition, the study demonstrated that 50% of the participants cited the media and hospitals as their source of information about cervical cancer and Pap smear, thus showing their important role in information dissemination and education. The authors highlight that a health education program about cervical cancer that incorporates media might be very impactful in that context [34]. Another study assessing cervical cancer and Pap smear awareness among undergraduate students showed that 71% of the students were aware of cervical cancer, however, only 33% were aware of Pap smear [36].

In this context, previous study carried out in Nigeria among sexually active woman demonstrated that only 26% of the respondents were aware of cervical cancer screening, and in addition, only 47% of the aware group knew that the test was to screen cervical cancer. The authors report an association between the educational status and the knowledge of Pap test, but not in relation to the test utilization [39].

Another study determining the level of awareness and uptake of cervical screening in Owerri, Nigeria has demonstrated as major finding that 52.8% of the respondents were aware of cervical screening and that 7.1% had ever done the test. The main reasons for not doing it were “no need for it“, lack of knowledge that it could be done locally, and fear and anxiety over a positive result [35].

Although our results demonstrate high IES throughout the studies (greater than 25%), meaning that the themes identified were consistent across the studies, the study quality assessments demonstrated that not all studies presented some of the assessed items or it was unclear, representing high and moderate risk of bias, respectively. Specifically, most studies did not have a representative sample and blinding of the outcome assessment. These limitations might have affected the quality of the information extracted from these studies, the size of the effect.

Findings from our study suggest though that while Insufficient Knowledge/ Lack of Awareness is a large limitation, to CPG adherence, interventions for improving adherence must be more comprehensive to further address Negligence/Misbeliefs and Psychological Reasons and well as the Time/Cost Constraints and Insufficient Infrastructure/Training. Given the high incidence of advanced stage cervical cancer in Africa, there is an urgent need for successful strategies for improving clinical practice guideline compliance. These findings support adopting a multifaceted approach, not only addressing provider education, but also patient education, cost, timing, infrastructure and psychosocial concerns. In addition, future work in this area should be performed to further understand the extent of the actual versus perceived psychosocial reasons and the validity of these reasons from the perspective of the patient. Secondly, further understanding of cost, time and infrastructure limitations, whether perceived or actual, should be undertaken.

Our data coincide with a previous study that reported implementing multifaceted approaches are needed to achieve optimal screening and treatment for cervical cancer [33]. Such approaches should involve improvement of health workers and community knowledge by implementing then evaluating education, and communication strategies, as well as establishing appropriate structure and guidelines, and developing new technologies [33]. Research has been conducted to evaluate alternative cervical cancer screening technologies in low-resource setting, which would require fewer resources and infrastructure, thus having the potential of increasing the feasibility of cervical screening implementation [43]. However, it has been demonstrated that technological interventions and innovations alone are not sufficient to improve cervical screening programs. Other concerns intrinsic to health systems in Africa include human resources concerns such as training, increasing demands on personnel, attrition, and skills mix. These should be addressed concurrently within a comprehensive workforce development strategy, alongside work to make the health care delivery system functional, otherwise the task shifting would be limited [44].

In conclusion, studies that have evaluated factors affecting healthcare provider clinical practice guideline adherence for Pap smears in Africa had high effect sizes, demonstrating that the themes identified were consistent across the studies. The studies suggest provider non-compliance factors include lack of awareness and knowledge about protocols, time and cost constraints, and lack of infrastructure or training on performing Pap smears. These results suggest that prevention initiatives should be multifaceted including education, resource needs assessments and improvement, Pap smear testing training as well as strategies on costing and practitioner time studies.

## Supporting Information

File S1 Study Protocol. (DOCX)

Table S1 PRISMA checklist. (DOC)

Table S2 PubMed Search Strategy. (DOCX)
