# Supplementary material for: Factors Affecting Compliance with Clinical Practice Guidelines for Pap Smear Screening among Healthcare Providers in Africa: Systematic Review and Meta-Summary of 2045 Individuals
Source: PLoS One. 2013 Sep 12;8(9):e72712. doi: 10.1371/journal.pone.0072712 (PMC3771969; doi:10.1371/journal.pone.0072712)
Supplement: File S1 — (DOCX) [file pone.0072712.s001.docx]

# STUDY PROTOCOL

# Factors Affecting Compliance with Clinical Practice Guidelines for Pap Smear Screening among Healthcare Providers in Africa: A Systematic Review and Meta-Summary

## BACKGROUND

There is a wide disparity of screening for cervical cancer between developed and developing countries.^(1-3)^ The World Health Organization (WHO) has already published clinical practice guidelines (CPGs) on cervical cancer, however, most developing African countries have not yet initiated widespread national cancer screening programs. A WHO report on cervical cancer screening in Sub-Saharan Africa noted that while this region was the most affected by cervical cancer, it has access to less than 5% of the global resources for cervical cancer prevention.^(4)^

In this context, healthcare providers behavior has been shown to have an important role in patient compliance.^(5-7)^ However, the factors affecting compliance with clinical practice guidelines for Pap smear screening among healthcare providers in Africa are still not well known. Thus, the objective of this systematic review is to identify factors affecting compliance with Clinical Practice Guidelines for pap screening among healthcare providers in Africa*.*

## METHODS

### Protocol and Registration

This systematic review will be reported in accordance with the Preferred Reporting Items for Systematic Review and Meta-Analyses (PRISMA) statement^(8)^.

### Eligibility Criteria

The following inclusion criteria will be considered: 1. Studies involving medical personnel; 2. Studies whose outcome measures including any factors that affect medical personnel from using a pap smear to screen for cervical cancer, 3. Studies conducted in Africa, and 4. Observational Studies. We will exclude studies that retrospectively analyzed clinical trial data, unpublished articles, dissertations, and abstracts without full text. In addition, manuscripts in languages other than English, French or Portuguese will not be included in the review.

### Information Sources

We will search the following electronic databases: MEDLINE (accessed by PubMed) MEDLINE, Scirus, Opengate and EMBASE. We will not use limits for language when searching the databases. The references of the included articles will be reviewed, as well as we will perform citation analysis of the included studies using Google Scholar, and also seek experts’ suggestions through email communications.

### Search

The initial search will comprise the following Mesh terms "*Vaginal Smears", and "Africa"* and the related entry terms.

**Reference lists**

We will manually check the reference list of all the studies identified by the above search strategy. Relevant studies shall be observed, and where appropriate, included in the review.

**Grey literature**

We will attempt to contact authors and other experts on the field for any relevant material.

### Study Selection

Titles and abstracts of the retrieved articles will be independently evaluated by 2 reviewers (E.A. and M.V.). Abstracts that do not provide enough information regarding the eligibility criteria will be kept for full-text evaluation. Reviewers independently will evaluate full-text articles and determine study eligibility. Disagreements will be solved by consensus and if disagreement persisted, we will seek a third reviewer’s opinion (A.J.).

### Quality of Studies

Quality assessment will be based on the Cochrane risk of bias tool (Review Manager version 5.1, Cochrane Collaboration). The following items will be considered: blinding of outcome assessment, incomplete outcome data, and selective reporting. In addition, we will add two more parameters: measurement methods that were not suitable or precise, and use of an unrepresentative sample^(9,10)^, as proposed by Friedemann C., et al (2012)^(11)^.

### Data Extraction

Two reviewers (E.A. and M.V.) will independently conduct data extraction and disagreements will be also solved by the third reviewer (A.J.). General characteristics of the studies will be collected, such as: year of publication, location and setting where the study took place, number of health care providers, health providers characteristics. In addition, we will collect from each study the factors cited as affecting compliance with CPGs for Pap smear screening among healthcare providers in Africa.

### Data Analysis

We will perform a descriptive analysis of factors affecting healthcare provider compliance with CPGs for Pap smear in Africa, described in the included studies. The factors affecting compliance will be grouped by similarity, and within each group, brief descriptions of the findings will be generated. To represent the magnitude of each finding, we will adapt the methodology proposed by Sandelowski M., et al (2007)^(16)^. Frequency effect sizes will be generated by dividing the number of studies citing a particular theme by the total number of studies in our final list. To represent the magnitude of each report, intensity effect sizes will be calculated. Specifically, for each study, the number of findings with a frequency effect size >25% will be divided by the total number of findings with frequency effect size >25%.  Additionally, for each study, the number of themes it cited will be divided by the total number of themes overall^(12)^.

**AKNOWLEDGMENTS**

The authors thank the Research on Research Group ([http://researchonresearch.duhs.duke.edu/](http://researchonresearch.duhs.duke.edu/)) for the templates for writing introduction and discussion sections of the manuscript^(13)^ as well as templates for Literature matrix, Duke University Health System^(14)^.

**REFERENCES**

1. Anttila A, Pukkala E, Söderman B, Kallio M, Nieminen P, et al. (1999) Effect of organised screening on cervical cancer incidence and mortality in Finland, 1963-1995: recent increase in cervical cancer incidence. International journal of cancer Journal international du cancer 83: 59-65.

2. Eddy DM (1990) Screening for cervical cancer. Annals of internal medicine 113: 214-226.

3. Gakidou E, Nordhagen S, Obermeyer Z (2008) Coverage of cervical cancer screening in 57 countries: low average levels and large inequalities. PLoS medicine 5.

4. World Health Organisation. (2002) Cervical Cancer Screening in Developing Countries: Report of a WHO consultation. Geneva; 3-50.

5. Lurie N, Slater J, McGovern P, Ekstrum J, Quam L, et al. (1993) Preventive care for women. Does the sex of the physician matter? The New England journal of medicine 329: 478-482.

6. McPhee SJ, Bird JA, Fordham D, Rodnick JE, Osborn EH (1991) Promoting cancer prevention activities by primary care physicians. Results of a randomized, controlled trial. JAMA: the journal of the American Medical Association 266: 538-544.

7. Cabana MD, Rand CS, Powe NR, Wu AW, Wilson MH, et al. (1999) Why don't physicians follow clinical practice guidelines? A framework for improvement. JAMA: the journal of the American Medical Association 282: 1458-1465.

8. Moher D, Liberati A, Tetzlaff J, Altman DG, The PRISMA Group (2009) Preferred Reporting Items for Systematic Reviews and Meta-Analyses: The PRISMA Statement. PLoS Medicine 6:1-6.

9. Altman DG. Systematic reviews of evaluations of prognostic variables. BMJ 2001;323:

224-8.

10. Heneghan C, Badenoch D. Evidence based medicine toolkit . 2nd ed. Blackwell Publishing,

2006.

11. Friedemann C, Heneghan C, Mahtani K, Thompson M, Perera R, Ward AM (2012) Cardiovascular disease risk in healthy children and its association with body mass index: systematic review and meta-analysis. BMJ 25:1-16.

12. Sandelowshi M, Barroso J, Voils CI (2007) Using Qualitative Metasummary to Synthesize Qualitative and Quantitative Descriptive Findings. Research Nursing Health 30:99-111.
13.     Shah J, Shah A, Pietrobon R (2009). Scientific writing of novice researchers: what difficulties and encouragements do they encounter? Academic Medicine: Journal of the Association of American Medical Colleges 84: 511-516.
14.     Pietrobon R, Guller U, Martins H, Menezes AP, Higgins LD, et al. (2004). A suite of web applications to streamline the interdisciplinary collaboration in secondary data analyses. BMC Medical Research Methodology 4: 29-29.
