# Supplementary material for: Factors Affecting Compliance with Clinical Practice Guidelines for Pap Smear Screening among Healthcare Providers in Africa: Systematic Review and Meta-Summary of 2045 Individuals
Source: PLoS One. 2013 Sep 12;8(9):e72712. doi: 10.1371/journal.pone.0072712 (PMC3771969; doi:10.1371/journal.pone.0072712)
Supplement: Table S2 — (DOCX) [file pone.0072712.s003.docx]

**Appendix 2:** Search strategy used for PubMed.

| **Search** | **Query** | **Items found** |
| --- | --- | --- |
| [#3](http://www.ncbi.nlm.nih.gov/pubmed/advanced) | Search #2 AND #1 | [346](http://www.ncbi.nlm.nih.gov/pubmed/?cmd=HistorySearch&querykey=19) |
| [#2](http://www.ncbi.nlm.nih.gov/pubmed/advanced) | Search (“Vaginal Smears”[Mesh] OR “Smear, Vaginal”[All Fields] OR “Smears, Vaginal”[All Fields] OR “Vaginal Smear”[All Fields] OR “Papanicolaou Test”[All Fields] OR “Test, Papanicolaou”[All Fields] OR “Papanicolaou Smear”[All Fields] OR “Smear, Papanicolaou”[All Fields] OR “Cervical Smears”[All Fields] OR “Cervical Smear”[All Fields] OR “Smear, Cervical”[All Fields] OR “Smears, Cervical”[All Fields]) | [20130](http://www.ncbi.nlm.nih.gov/pubmed/?cmd=HistorySearch&querykey=18) |
| [#1](http://www.ncbi.nlm.nih.gov/pubmed/advanced) | Search (("africa"[Mesh] OR "africa"[All Fields]) | [197158](http://www.ncbi.nlm.nih.gov/pubmed/?cmd=HistorySearch&querykey=15) |
